# The effect of prolactin on immune cell subsets involved in SLE pathogenesis

**DOI:** 10.3389/fimmu.2022.1016427

**Published:** 2022-10-28

**Authors:** Maria Victoria Legorreta-Haquet, Paola Santana-Sánchez, Luis Chávez-Sánchez, Adriana Karina Chávez-Rueda

**Affiliations:** Unidad de Investigación Médica en Inmunología (UIM) en Inmunología, Hospital de Pediatría, Centro Médico Nacional (CMN) Siglo XXI, Instituto Mexicano del Seguro Social, México City, Mexico

**Keywords:** prolactin, SLE, immune cells, autoimmune disease, immune response

## Abstract

The higher frequency of autoimmune diseases in the female population compared to males suggests that certain hormones, such as prolactin (PRL), play a role in determining the prevalence of autoimmunity in women, particularly during childbearing age. PRL can act not only as a hormone but also as a cytokine, being able to modulate immune responses. Hyperprolactinemia has been implicated in the pathogenesis of various autoimmune diseases where it may affect disease activity. One of the conditions where PRL has such a role is systemic lupus erythematosus (SLE). PRL regulates the proliferation and survival of both lymphoid and myeloid cells. It also affects the selection of T-cell repertoires by influencing the thymic microenvironment. In autoimmune conditions, PRL interferes with the activity of regulatory T cells. It also influences B cell tolerance by lowering the activation threshold of anergic B cells. The production of CD40L and cytokines, such as interleukin IL-6, are also promoted by PRL. This, in turn, leads to the production of autoantibodies, one of the hallmarks of SLE. PRL increases the cytotoxic activity of T lymphocytes and the secretion of proinflammatory cytokines. The production of proinflammatory cytokines, particularly those belonging to the type 1 interferon (IFN) family, is part of the SLE characteristic genetic signature. PRL also participates in the maturation and differentiation of dendritic cells, promoting the presentation of autoantigens and high IFNα secretion. It also affects neutrophil function and the production of neutrophil traps. Macrophages and dendritic cells can also be affected by PRL, linking this molecule to the abnormal behavior of both innate and adaptive immune responses.This review aimed to highlight the importance of PRL and its actions on the cells of innate and adaptive immune responses. Additionally, by elucidating the role of PRL in SLE etiopathogenesis, this work will contribute to a better understanding of the factors involved in SLE development and regulation.

## Graphical Abstract

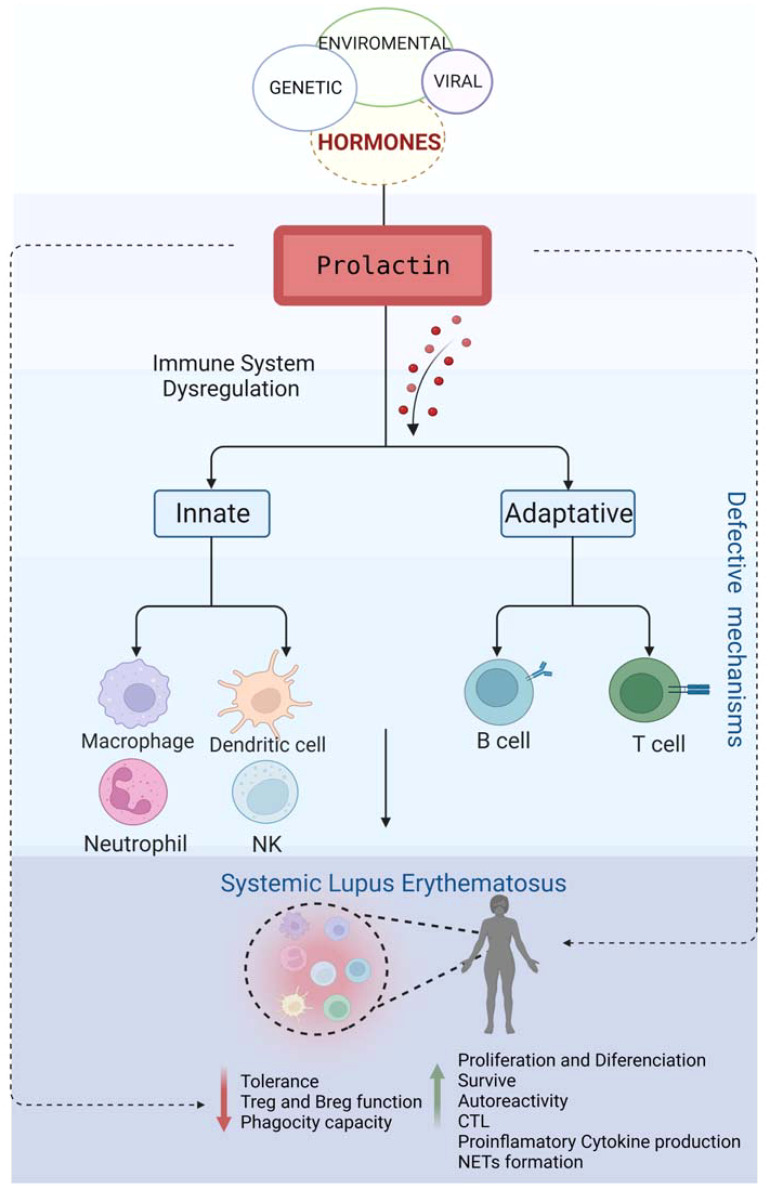

## Introduction

Prolactin (PRL) is a globular protein comprised of four α-helices. Consisting of 199 amino acids, it has a molecular weight of 23 kDa. Its monomeric hormone form contains two PRL receptor binding domains (1). PRL can present in several isoforms due to posttranslational modifications. Apart from the most prevalent monomeric structure, large PRL and macroprolactin (“big-big”) are the two other commonly detected isoforms. Of these, the monomeric form is the most biologically active. PRL is secreted in the pituitary gland, but it can also be produced in extra-pituitary sites. The decidua, ovary, prostate, mammary glands, adipose tissue, brain, and immune cells can also produce the hormone (2). Pituitary PRL secretion is regulated by hypothalamic factors, such as thyrotropin-releasing factor and dopamine, *via* the pituitary portal circulation (3).

Various biological functions have been ascribed to PRL, but its role in the regulation of the immune system is by far the most controversial. PRL exerts its biological effects *via* interacting with a canonical receptor, which is, perhaps surprisingly, a member of the cytokine receptor superfamily. The receptor-ligand interaction triggers a number of signaling pathways, including the P13K/Akt, MAPK, and JAK/STAT signaling, which are involved in the proliferation, differentiation, and survival regulation of immune cells (1, 4).

The surprisingly wide tissue distribution of PRL receptor (PRLR) provides an explanation for the increasing number of biological roles attributed to PRL. While the main function of prolactin is to stimulate the proliferation and differentiation of mammary cells required for lactation, studies in animal models and humans have described more than 300 different actions attributed to PRL (1).

The structure of the human PRLR contains multiple short motifs within its intracellular domain, allowing it to simultaneously interact with several kinases and associated signaling pathways. The resulting combination of distinct cellular responses may partly explain the versatility of PRL actions (5).

The main isoform of PRLR in humans is a long protein composed of 598 amino acids that binds at least three ligands: PRL, placental lactogen, and growth hormone. The presence of two receptor binding sites on a monomeric PRL enables the hormone to bind two receptors at the same time, resulting in a receptor dimerization-driven signal transduction (6).

The extracellular domain of PRLR is analogous to type III fibronectin and consists of approximately 200 amino acids. It is divided into two subdomains of approximately 100 amino acids each. The first subdomain contains two disulfide bridges (Cys12-Cys22 and Cys51-Cys62), while the second one has a pentapeptide motif referred to as WS (Trp-Ser-aa-Trp-Ser). The transmembrane domain is composed of 24 amino acids, while the intracellular domain differs in size and composition depending on the receptor isoform (7). The long form of PRLR induces the activation of both Jak-2 and Fyn, while the intermediate form activates Jak-2 only. These functional differences appear to be due to structural changes since Y587 residue contributing to the activation of Stat-5 and SHP-2 in long PRLR is absent in the intermediate form. Interestingly, PRLR expression has been detected on the surface of monocytes, macrophages, T cells, NK cells, granulocytes, thymic epithelial cells, and B cells (8).

The higher incidence of certain autoimmune diseases in women suggests that female hormones are important risk factors in the etiopathogenesis of these conditions. The influence of hyperprolactinemia on the development of various autoimmune diseases, such as rheumatoid arthritis (RA), multiple sclerosis (MS), autoimmune thyroid disease, uveitis, and systemic lupus erythematosus (SLE), has been widely discussed but remains controversial. SLE patients have higher plasma/serum PRL levels, but this varies by region (9). More research is needed to determine the role of PRL in autoimmune disease because the mechanism(s) underlying this association are not fully understood, despite the extensive documentation of the PRL-mediated regulation of immune cells.

## Prolactin and SLE: Adaptive immune response cells

### The effect of PRL on T cells

Once formed in the thymus, mature T cells migrate to peripheral lymphoid organs, where the final stages of their development occur. This process includes cell proliferation, migration, receptor rearrangement, activation, and, ultimately, cell death. Hormones have been shown to control thymic physiology, influencing the development of T-cells (10). This control consists of endocrine, paracrine, and autocrine pathways acting on cells of the thymic microenvironment and thymocytes themselves and being able to influence the selection of the T-cell receptor repertoire (11). Developing thymocytes constitutively produce PRL (12). The autocrine/paracrine secretion of the hormone is an important factor in the survival and proliferation of double-negative early T-cell precursors (CD25+CD4-CD8-). As PRL also induces the expression of interleukin IL-2 receptor in these cells, it also influences their maturation into CD4+ CD8+ T lymphocytes (13).

Nb2-11 is a clone of the Nb-2 rat lymphoma line, which was derived from a transplant of lymphoma following prolonged estrogen treatment. The cells are of the pre-T cell origin, and their proliferation depends on mammalian lactogens, such as PRL (14). Their activation *via* the PRLR-driven phosphorylation of ZAP-70 leads to the decreased expression of CD3. In contrast, the activation of the T cell receptor (TCR) complex *via* anti-CD3 antibodies prevents T cell mitogenic stimulation through PRLR. Therefore, at least in this clone, PRL and TCR-driven activation appear functionally related (15). However, it is critical to recognize the limitations of using transformed cells. Although they are an important part of the research, their physiological relevance might be inferior to that of primary cells.

Although the literature is not conclusive on the effect of PRL on the immune response, several articles have substantiated the immunomodulatory effect of this hormone. Pellegrini et al. have been the first to demonstrate the expression of PRL and its receptor in peripheral blood mononuclear cells, including purified T cells (16). Subsequent reports have indicated that PRL could induce the expression of IL-2 receptors. This process may represent an autocrine regulation loop, as cells of the immune system can produce PRL themselves. Additionally, evidence has indicated that an optimal concentration of PRL favors a normal immune function, while abnormally low or high PRL levels can result in a partially immunocompromised state (17).

PRL promotes cytokine production and expression of activation markers in normal CD8+ T cells stimulated with mitogens (18). Matera et al. have reported PRLR expression in activated CD8+ T cells. The addition of exogenous PRL (6–20 ng/ml) to the culture media of these activated cells increased their IFN-γ secretion. In contrast, the addition of PRL at higher concentrations (200 ng/ml) had the opposite effect, reducing IFN-γ secretion. The cytotoxic activity of CD8+ T cells also showed a similar, dose-dependent modulation. While physiologic levels of PRL increased cytotoxic activity, higher concentrations, compatible with hyperprolactinemia, significantly suppressed it (19).

However, some authors have suggested that PRL inhibits cytokine production. Ellah et al. have reported that serum levels of proinflammatory cytokines (IL-1β, IL-6, and tumor necrosis factor TNF-α) and the amount of peripheral regulatory T cells (Tregs) were significantly higher in diabetic rats. In this model, the administration of metoclopramide, an inducer of PRL secretion, led to a significant decrease in the serum levels of these proinflammatory cytokines. Indeed, metoclopramide has been used as an immunomodulatory agent due to its ability to restore cellular immune function through increased PRL secretion (20).

It has been reported that PRL can also influence CD4+ T cell responses *via* the modulation of T-bet. Low doses of PRL induce the expression of T-bet, while high doses suppress it (21). Additionally, low doses of PRL (10–30 ng/ml) promoted the secretion of helper effector T cells (Th)1-type cytokines (IFN-γ and IL-12), while the little effect was observed at high doses (more than 100 ng/ml). This might indicate that low doses of PRL promote the secretion of Th1 cytokines through this mechanism (22). It appears that PRL also acts on human keratinocytes by increasing the production of CXCL9, CXCL10, and CXCL11 and driving the preferential recruitment of Th1 cells (23). The addition of exogenous PRL significantly increases the expression of CD69 on CD8+ T cells and upregulates CD25 on peripheral blood mononuclear cells (24). In line with these observations, the addition of antibodies blocking PRL to the culture media results in reduced CD69 and CD154 expression on TCD4+ cells (25).

### The relationship between PRL, T cells, and SLE

SLE is an autoimmune disease characterized by altered homeostasis of both the innate and adaptive immune systems. It is characterized by increased apoptosis, hyperactivation of T cells, increased autoantibody production, and immune complex deposition. The disease primarily affects women of childbearing age and is caused by a complex interaction of genetic, environmental, and hormonal factors, leading to the breakdown of immunological tolerance.

Th cells are derived from naïve CD4+ T cells that differentiate into different cell subtypes after antigenic stimulation-induced maturation. This process depends on complex interactions with antigen-presenting cells, costimulatory molecules, and cytokine signaling. CD4+ T cells can differentiate into distinct phenotypes, including Th1, Th2, Th9, Th17, Th22, regulatory, and follicular T cells. A subset of them becomes memory T cells. Furthermore, there are other unconventional T cells; for example, those expressing γδ T cell receptors or those restricted by CD1. Each of these subtypes expresses a unique transcriptional profile (26).

In several autoimmune diseases, including SLE, symptoms worsen during pregnancy, while in other diseases, for example, MS, RA, uveitis, and thyroiditis, the illness will temporarily improve. This opposing effect of pregnancy is probably due to a change in the balance between Th1- and Th2-type immune responses. This change occurs to avoid the immunological rejection of the fetus and increase the production of maternal antibodies providing passive immunity to the fetus. Thus, autoimmune conditions driven by a primarily Th1 response benefit from this deviation, while the switch to Th2 predominance worsens others, including SLE, due to the enhanced autoantibody production (27–30). Moreover, maternal antibodies can have a significant impact on fetal health. Fetal growth and development in SLE patients might be threatened by disease activity, abnormality of maternal renal function, antiphospholipid antibodies, and anti-SSA/Ro and anti-SSB/La antibodies. Antiphospholipid antibodies cause placental insufficiency, intrauterine growth restriction, and fetal death (31, 32).

**Figure 1:**
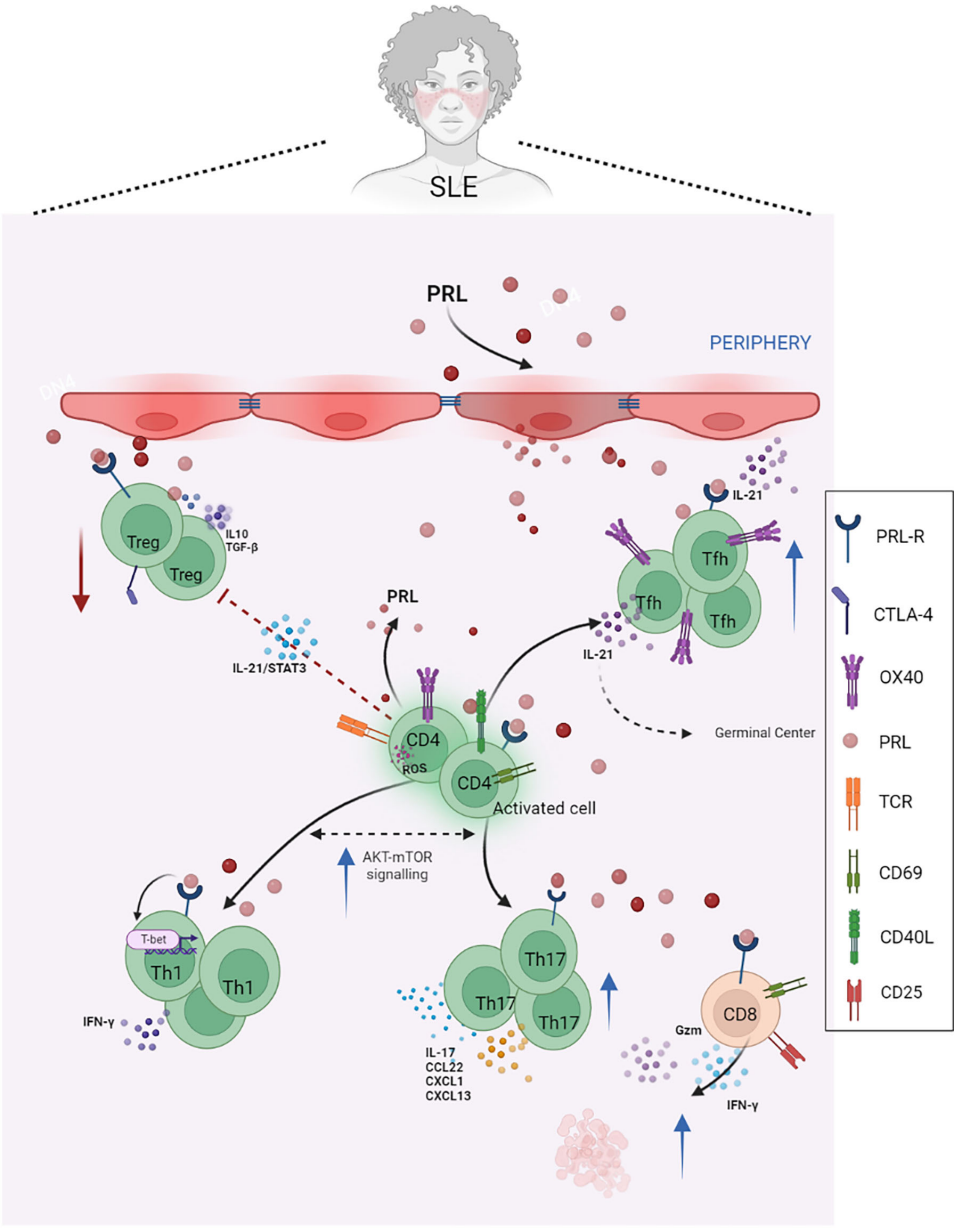
PRL in T cells and SLE. PRL exacerbates the mechanisms of activation and differentiation of T helper lymphocytes, mainly towards Tfh, Th17, and Th1 lymphocytes, and reduces the functional capacity of regulatory T lymphocytes, which are already reduced in SLE. In patients with SLE, IL-21 blocks Treg differentiation and function by being activated in a STAT3-dependent manner. These patients also have a higher percentage of circulating CD4+ OX40+ T cells and Th17 cells, as well as increased serum levels of IL-17, which correlates with disease activity. PRL contributes to the secretion of proinflammatory cytokines, such as IFNγ, and the expression of activation molecules, such as CD69 and CD40L, as well as the cytotoxic activity of CD8+ T cells, favoring the availability of autoantigens and, therefore, the phenomenon of autoimmunity (Created with BioRender.com).

Molecular and metabolic alterations causing abnormal T cell function have been described in both SLE patients and lupus-prone mouse models. In SLE patients, the increased AKT-mTOR signaling pathway was reported in T cells, favoring the differentiation of Th1 and Th17-cell lineages (33, 34). Greg M. Delgoffe et al. have demonstrated that mTOR activation is necessary for Th1, Th2, and Th17 effector T cell differentiation. Furthermore, even under fully activating conditions, T cells lacking mTOR differentiated into Foxp3+ regulatory cells (35). Additionally, T cells from SLE patients appear chronically active, and the hyperactivity in TCR signaling causes increased reactive oxygen species (ROS) secretion as a consequence of mitochondrial hyperpolarization and ATP depletion (36) (**Figure 1**).

The mTOR signaling pathway is closely related to SLE pathogenesis. The improved understanding of immunoregulation and completion of more clinical trials is expected to define an mTOR-targeted therapeutic intervention as a new target for precision medicine for SLE treatment. More extensive and future research with larger trial populations is required to determine its efficacy and tolerability.

SLE is a highly complex disease. Its pathogenesis involves multiple factors related to the immune system. However, there is a growing recognition of the role of T cells in its pathogenesis.

Alterations in cytokine production and T-cell signaling have been shown in SLE, resulting in both abnormal T-cell differentiation and excessive B-cell activation. STAT proteins are essential for the differentiation of specific cell lineages and have a fundamental role in the development of autoimmune diseases (37). In patients with SLE, there is a decreased IL-2 production, reducing the phosphorylation of JAK-3 and STAT-5 (38). The level of basal STAT signaling appears to correlate with reduced cytokine responses, increasing disease activity and severity (39).

Using single-cell transcriptomic studies from kidney samples, Arazi et al. have reported an abundant presence of NK cells and cytotoxic T cells in renal lymphoid nodules of SLE patients. These cells were the main sources of IFNγ and cytolytic molecules. Interestingly, the interferon response of these infiltrating lymphocytes mimicked the same responses observed in lymphocytes derived from peripheral blood.

In SLE patients, the depletion of PRL produced by T cells with anti-PRL antibodies results in an important decrease in CD69 and CD154 expression. This indicates that PRL participation is a step between the trigger (antigen presentation) and costimulatory signal, which is a relevant step in the development of autoimmunity, particularly in SLE (40).

### Regulatory T cells

Tregs represent a subset of T cells that express the IL-2 receptor α chain (CD25). Their deficiency in number and/or function has been implicated in the breakdown of self-tolerance and the development and progression of autoimmune diseases. These cells have been initially identified due to their ability to prevent the onset of systemic autoimmune diseases in thymectomized mice (41). It is now known that Treg cells are essential for maintaining self-tolerance by suppressing the activation and expansion of T cells expressing potentially autoreactive T cell receptors. Regulatory T cells function *via* both cell-cell interactions and secretion of anti-inflammatory cytokines, especially IL-10 and TGF-β (42). Several mechanisms have been described in the thymic microenvironment that influences the function of Treg cells. For example, in SLE patients, IL-21 blocks Treg differentiation and function by activating mTORC1 and mTORC2 in a STAT3-dependent manner. IL-21 also inhibits GATA-3 and CTLA-4 expression by regulatory T cells (43) (**Figure 1**).

Some immune responses differ between men and women. For example, females produce stronger inflammation protecting them against infection. However, this renders women more susceptible to the development of autoimmune diseases. Factors contributing to these differences are primarily linked to the X chromosome and affect the expression of a key regulator of Treg numbers and function, *FOXP3* (44).

Legorreta et al. have reported that SLE patients have reduced Treg cell numbers, and the remaining cells show functional deficits. Furthermore, PRL reduces the suppressor effect of Treg cells, as these cells constitutively express PRLR. Additionally, the action of the hormone favored the secretion of IFNγ, resulting in a proinflammatory microenvironment (45). This finding could point to a link between the PRL signaling pathway and SLE progression (**Figure 1**).

PRL secretion caused by psychological stressors has been described as a facilitating factor in the onset of intestinal inflammation. This appears to be linked to the modulation of IL-6 and IL-23 secretion by dendritic cells. This modulation, in turn, alters the Treg cell phenotype towards Foxp3+ cells producing IL-17 and TNF-α (46).

### Follicular helper T cells

Follicular helper T (Tfh) cells represent a heterogeneous subtype of CD4+ lymphocytes inducing the differentiation of B cells into plasma and memory cells (47). Tfh cells interact with B cells to promote isotype switching and antibody production. However, this mechanism can also induce autoantibody production in autoimmune diseases. Tfh cell development is necessary for adequate antibody response, mainly through the activation and maintenance of the germinal center (GC) response. Tfh cells express B-cell lymphoma transcription factor 6 (BCL6) (48) and inducible costimulator (ICOS), which are two molecules essential for the sustained interaction between T and B cells. This interaction induces the expression of key factors necessary for B cell activation, such as death protein 1 (PD-1) and CD40L (49).

It is known that the amount of circulating Tfh cells is increased in SLE patients. Furthermore, the number of these cells in the blood shows a positive correlation between autoantibody titers and disease activity (50).

These cells have been shown to contribute to SLE pathogenesis through the ICOS-B7RP-1 pathway in NZB/NZW F1 mice. The blockade of this pathway induces a reduction in both B cell numbers and GCs, improving the manifestation of RA and SLE in this murine model (51). Aleman et al. have reported that PRL increased the number and activation of IL21-producing OX40+ Tfh cells. These cells promoted the formation of GCs, interfered with tolerance induction, and facilitated the generation of autoreactive plasma cells, with the consequent autoantibody secretion (52). In SLE patients, a higher percentage of CD4+ OX40+ T cells has also been shown to indicate increased disease activity (53) (**Figure 1**).

### Th17 lymphocytes

Th17 cells represent a subset of T cells with proinflammatory properties. The initial differentiation of Th17 cells from naive CD4+ T cells is dependent on IL-1β, IL-6, and TGFβ, while their further expansion is promoted by IL-23 and IL-21 (54). Th17 cells produce IL-17A, IL-17F, IL-21, and IL-22 and can increase the production of proinflammatory cytokines, including TNF-α and IL-6. This mechanism increases inflammation and promotes autoimmunity by inducing the proliferation, maturation, and recruitment of neutrophils, macrophages, and lymphocytes (55).

Previous studies have reported elevated levels of IL-17 in the blood and tissues of patients with inflammatory bowel disease and psoriasis, suggesting that this cytokine can promote deleterious inflammatory responses. High serum and plasma IL-17 levels have also been observed in SLE patients (56) and murine SLE models (57). Furthermore, the amount of circulating IL-17 producing CD4+ T cells correlated with disease activity. Plasma levels of IL-6, a cytokine that promotes the development of Th17 cells, have been higher in SLE patients than in healthy subjects. This mechanism might be a relevant factor in SLE development (58). Certain evidence has indicated that hyperprolactinemia alters the balance between Th1, Th2, and Th17 cells (59). For example, hyperprolactinemia has been linked to increased levels of CCL22, CXCL1, and CXCL13. These chemokines are associated with Th2 and Th17 responses and attract B-cell migration *in vivo*. However, increased chemokine levels in the serum of hyperprolactinemia patients have not been demonstrated (60). On the other hand, in a murine model of psoriasis, intraperitoneal administration of PRL increased Th17- and Th1-type cytokine mRNA abundance in the skin treated with imiquimod (TLR7 agonist). This mechanism increased inflammation and exacerbated psoriasis-like symptoms (61). Meanwhile, findings in experimental autoimmune encephalitis (EAE) have shown, on the one side, that PRL does not appear to play a central role in the expression of full disease severity. However, in PRL or PRLR deficiency conditions, a delay in the onset of EAE symptoms has been observed, which has been associated with a delay in the establishment of Th1 and Th17 responses (62). Yakamura et al., for their part, have highlighted the possible pathogenic role of PRL in EAE by reporting that APCs that infiltrate late EAE lesions could secrete PRL and that it played a critical role in the induction of eomesodermin-positive CD4+ T cells, which are mediators of persistent neuroinflammation in late-stage EAE (63). To varying degrees, PRL appears to promote autoimmunity.

PRL enhances the production of CCL20 by keratinocytes. Given that CCL20 causes the preferential recruitment of Th17 cells, this mechanism might be part of the puzzle of autoimmune phenomena (64). Thus, although there is a clear relationship between PRL, Th17 cells, and autoimmunity, the underlying mechanisms remain to be fully elucidated.

In SLE, IL-17 promotes inflammation by inducing the local production of chemokines and cytokines and increases the production of autoantibodies (65). Both the number of circulating Th17 cells and serum IL-17 levels have been reported to increase in SLE, and serum levels of IL-17 show a significant association with proteinuria and disease activity (66) (**Figure 1**). Th17 cell potent proinflammatory activities might contribute to several SLE pathological pathways, and PRL could be involved in this axis. For example, the clear interplay between Th1 and Th17 cell lineages is evident, as is the interaction between PRL and Th1, suggesting that PRL may have a role in this axis related to SLE pathogenicity.

### The effect of PRL on B cells

B cells, apart from producing antibodies, are important antigen-presenting cells (APCs) in T cell activation. The diversity of the B-cell receptor (BCR) repertoire allows the recognition of numerous pathogens. However, when B-cell tolerance is impaired, the same mechanism generates potentially autoreactive B cells, which are immature B cells with the capacity to recognize self-antigens.

During B-cell maturation, the percentage of autoreactive B cells gradually decreases. This is achieved through BCR editing, clonal deletion, and anergy induction (67). Immature B cells leave the bone marrow and migrate to the spleen as transient B cells. Some of these may escape negative selection and can mature as marginal zone B cells (MZ) or follicular B cells (Fo) and undergo germinal center affinity maturation. MZ and Fo B cells show different antibody production kinetics, with MZ cells showing faster and longer-lasting antibody secretion. Both MZ and Fo B cells participate in the anti-DNA responses observed in murine models of SLE (68). B cells also play an important role in SLE pathogenesis *via* the production of autoantibodies that induce inflammation and cause immune complex deposition.

Peeva et al. have demonstrated that prolactin alters B cell development and maturation, causing a decrease in the number of transitional B cells and an increase in the number of mature follicular and marginal zone B cells, which breaks B cell tolerance and generates a lupus-like phenotype in non-susceptible mice with a significant increase in serum anti-DNA antibody titers and IgG deposits in the glomeruli (69). B cells produce PRL in bone marrow, highlighting the importance of the hormone in B-cell development (70, 71). The highest PRLR expression was detected in transitional B cells, and the expression levels of the receptor have also been associated with autoreactivity (72). B cells developing during hyperprolactinemia become short-lived plasma cells. Thus, long-term autoantibody production requires the ongoing maturation of transient DNA-reactive B cells into Fo B cells (68).

In murine animal models, hyperprolactinemia inhibited the BCR-mediated apoptosis of transient B cells in a CD40-CD40L-dependent process. This mechanism prevented the BCR-mediated clonal deletion of autoreactive cells. This altered, dysregulated receptor editing can lower the threshold for the activation of anergic B-cells and renders B-cell tolerance induction ineffective (72).

### The relationship between PRL, B cells, and SLE

**Figure 2:**
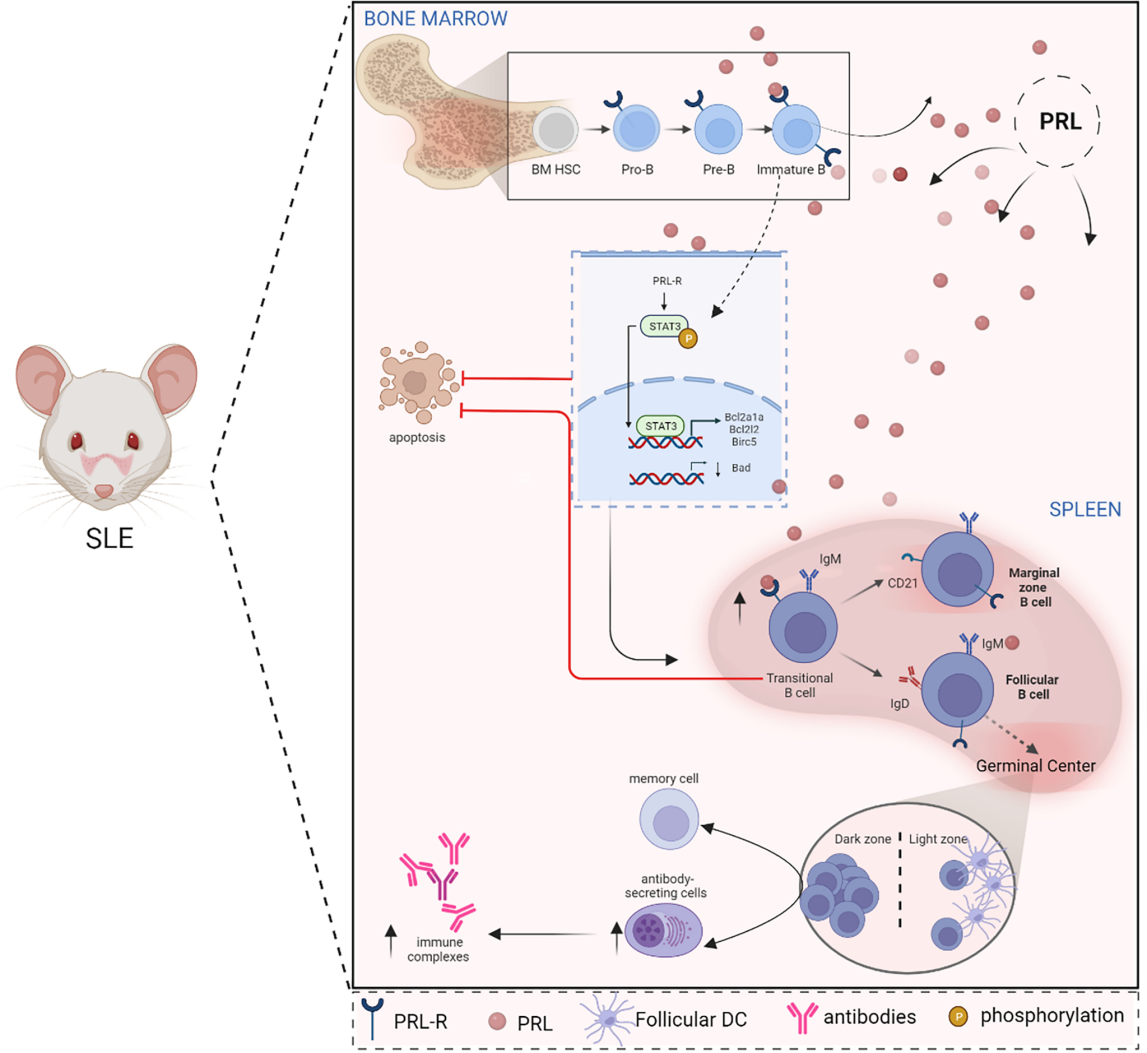
PRL alters B cell ontogeny in SLE. In SLE, there is a relationship between PRL and the ontogeny of B cells since the PRL receptor is present in the different stages of maturation of these cells in the bone marrow (Pro-B, Pre-B, and immature B cells) as in the spleen (transitional cells). PRL signaling in immature and transitional cells influences the evasion of clonal elimination mechanisms (apoptosis), promoting the development of autoreactive B cells. In immature cells, PRL favors STAT3 signaling and increases the expression of anti-apoptotic genes, such as *BCL2A1A, BCL2L2*, and *BIRC5*, while pro-apoptotic genes, such as *BAD*, are downregulated. The development and survival of autoreactive B cells correlate with the formation of autoantibody-producing cells and the consequent formation of immune complexes that increase the clinical manifestations of SLE (Created with BioRender.com).

B cell activation *via* the PRLR signaling promotes the phosphorylation and positive regulation of STAT3 and the anti-apoptotic genes *BCL2A1A, BCL2L2*, and *BIRC5*. Through these mechanisms, PRL participates in the onset of SLE by rescuing cells destined for clonal deletion. The activation of PRLR could also affect clones of immature autoreactive B cells through STAT3 activation and the transcriptional regulation of apoptosis resistance-related genes in an animal SLE model (73). This decreased apoptosis may be due to the upregulation of anti-apoptotic *BCL-XL* gene and a concomitant decrease of Bad expression caused by PRL in the MRL/lpr model (74). Furthermore, in murine models, hyperprolactinemia is associated with the pronounced secretion of anti-DNA IgG antibodies, immune complex formation, glomerulonephritis, and accelerated mortality (75) (**Figure 2**).

Gene expression studies have described the presence of B cells and follicular T-like cells in 50% of kidney biopsies from patients with lupus nephritis. In contrast, such cells were absent in healthy control samples. This reinforces the idea that kidney damage in SLE is induced by an *in situ* immune response (76). PRL inhibits B cell tolerance induction, increases proliferative response to antigens and mitogens, and increases the production of cytokines and autoantibodies (77), which are related to SLE.

## Prolactin and SLE: Innate immune response cells

### The effect of PRL on dendritic cells

Dendritic cells (DCs) are commonly classified into conventional and plasmacytoid DCs (pDCs). Conventional DCs are professional antigen-presenting cells participating in the activation and differentiation of T cells. In contrast, pDCs are poor activators of T cells due to their low expression of MHC class II and costimulatory molecules.

In adult animals, PRL deprivation has repeatedly induced immunodeficiency and prevented the development of autoimmune diseases (78). By increasing the expression of MHC II molecules and CD40, PRL can modulate the differentiation and maturation of DCs in both mice and humans (79). PRL-treated DCs also produce more proinflammatory cytokines, such as IL-12, TNF-α, and IL-1β, but not IL-6 or IL-10 (80).

In women suffering from hyperprolactinemia, the number and percentage of circulating DCs and pDCs are reportedly lower (81), yet it could be controversial regarding PRL relevance to DCs function. However, in a study that has looked at the effect of granulocyte-macrophage colony-stimulating factor and PRL on the maturation of DCs from blood monocytes in the absence of serum, the physiological concentration of PRL had a synergistic effect on the maturity and function of DCs (82). Another study has found that the presence of PRL in thymocyte cultures did not increase the number of DCs but stimulated their differentiation (78). PRL may influence physiological and pathological immune responses by altering DC viability, phenotype, stimulatory capacity, and cytokine expression (79).

### The relationship between PRL, dendritic cells, and SLE

The abnormal regulation of both innate and adaptive immune responses is a fundamental feature of SLE. One of the proposed mechanisms in SLE development is the alteration of the number, phenotype, and function of DCs. Zhou et al. have compared pDC phenotype and function in various SLE-prone mouse models. They reported pDC hyperactivity in some lupus strains resulting in high IFNα expression in response to stimulation *via* TLR7 or TLR9 (83). On the other hand, pDCs were absent in BXSB-RTD mice prone to developing lupus, leading to a reduction in symptoms and disease severity (84).

Approximately 50% of SLE patients carry a type I IFN gene signature. With pDCs being the main producers of type I IFN, it is reasonable to assume that they are the main contributors to this IFN gene signature, although contributions from other cell populations, including monocytes and neutrophils, cannot be entirely excluded (85). IFN-α can activate lymphocytes, DCs, and natural killer (NK) cells, potentially leading to the breakdown of immune tolerance.

In SLE patients, IFN-α can promote the transformation of monocytes into pDCs, which can recognize antigens and continuously produce IFN-α to activate lymphocytes, DCs, and NK cells. In mouse models, antinuclear antibody production is dependent on endosomal TLRs, which bind to dsDNA or ssRNA (86). In SLE patients, stimulation *via* the TLR9 ligand (using CpG) can activate pDC-like DCs to produce a large amount of type I IFN. More than half of SLE patients have high titers of anti-DNA antibodies against double-stranded DNA (dsDNA), and the titer of these antibodies correlates with disease activity. In addition to causing nephritis, anti-DNA antibodies can result in the formation of immune complexes. These, in turn, alter the induction of cytokines. The production of type 1 IFN by pDCs has been implicated in this process again (87). In SLE, apoptotic material is a source of endogenous nucleic acids that can be potent inducers of inflammatory cytokines *via* the nucleic acid-sensing pattern recognition receptors, TLR7 and TLR9. It has been proposed that the aberrant expression of TLR7 plays a key role in autoreactivity in both murine models and human autoimmunity (88). However, while the loss of TLR9 reduces antinuclear antibodies, it exacerbates the disease in some SLE models (89). There are some reports showing that PRL promotes autoimmunity *via* modulating the activation of lymphocytes and pDCs (27, 77).

A regulatory feedback loop has been described between pDCs and regulatory B cells (Breg). In this loop, IFN-α secreted by pDCs induces Breg cells to produce IL-10. IL-10, in turn, reduces IFN-α production by pDCs, providing negative feedback. However, this mechanism appears to be ineffective in some disorders, including SLE. The result is the hyperactivation of pDCs that cannot induce adequate differentiation of CD24+CD38hi Breg cells (90).

**Figure 3:**
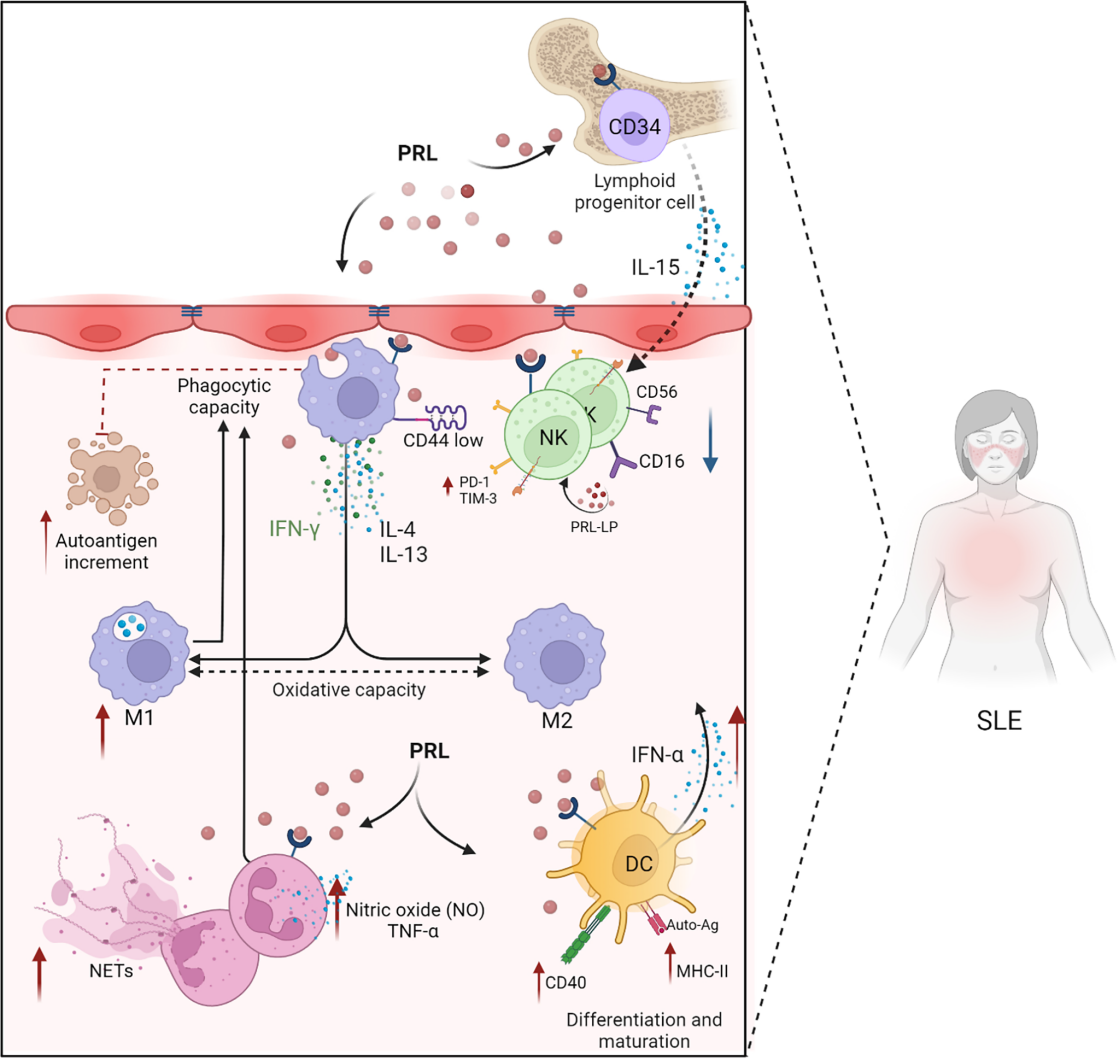
PRL in the innate immune response. PRL favors the development and progression of SLE through its intervention in the cellular mechanisms of the innate immune response (neutrophils, macrophages, dendritic cells, and NK cells). PRL exacerbates the mechanisms of cell activation and differentiation. PRL participates in the differentiation of NK cells through the modulation of IL-15 and the differentiation and maturation of dendritic cells by increasing the expression of CD40 and MHC-II. PRL decreases the phagocytic capacity of neutrophils, preventing the elimination of apoptotic bodies that could act as self-antigens capable of exacerbating the immune response, allowing autoimmunity to progress. In SLE, there is dysregulation between the M1 and M2 macrophages with a predominance in proinflammatory M1 macrophages, and PRL is probably involved in these mechanisms by favoring the presence of oxidative and proinflammatory molecules, such as IFN-γ, IFN-α, and TNF-α (Created with BioRender.com).

In SLE patients, IFN-α induces the differentiation and activation of DCs from monocytes, which can activate autoreactive T and B cells, and IFNs produced by pDCs start a self-perpetuating feedback loop that drives autoantibody production in SLE (91). Changing and activating DC subsets is a necessary first step in activating self-reactive lymphocytes, producing pathogenic autoantibodies, and starting a chronic inflammatory response. This reinforces the notion that PRL could participate in the maturation and differentiation of DCs, potentially promoting the presentation of autoantigens and elevated levels of IFN-α production. Through these mechanisms, PRL might have a fundamental role in SLE development (**Figure 3**).

### The effect of PRL on neutrophils

Neutrophils are the most abundant cells of the innate immune system. They are derived from the differentiation of hematopoietic stem cells (HSCs) and represent the first line of defense in the initiation of inflammatory responses to infections. They exert an antimicrobial action *via* phagocytosis, degranulation, and the production of neutrophil extracellular traps (NETs). NETs are extracellular networks of DNA, granule components, histones, and cytoplasmic proteins. They contribute to antimicrobial defense by trapping pathogens (92, 93). The formation of NETs is implicated in the development of various diseases, including SLE. In neutrophils, PRL induced an oxygen-free radical priming response, favoring pro-inflammatory activity (94).

### The relationship between PRL, neutrophils, and SLE

In sterile cell activation assays, macrophages from healthy donors could degrade PMA-induced NETs without secreting proinflammatory cytokines (95). However, in autoimmune diseases such as SLE, the degradation of NETs appears altered (96). The presence of spontaneous NETosis was described in lupus and has been linked to the presence of anti-DNA antibodies, chronic activation of pDCs *via* TLR9, and the induction of IFN-α secretion. Antimicrobial peptides released by neutrophils, such as LL-37, play a role in the activation of this pathway. The complexes these peptides form with autologous DNA induce the secretion of autoantibodies directed against antimicrobial peptide-DNA complexes (97).

Neutrophils are dysregulated in SLE and appear to produce reduced amounts of ROS during phagocytosis (98, 99). This mechanism contributes to the defective clearance of apoptotic cells. This defective clearance, in turn, leads to the production of autoantibodies.

Although no clear relationship has been established between PRL secretion and neutrophil defects in SLE, it is known that PRL alters the phagocytosis and intracellular killing of pathogens by neutrophils (100). The migration of neutrophils also appears affected in patients with hyperprolactinemia (101). Furthermore, the production of nitric oxide (NO) and TNF-α by neutrophils is altered in rats treated with PRL (94). Therefore, there are many mechanisms through which neutrophils could contribute to the progression of inflammation in SLE and regulate the function of other immune cells (**Figure 3**).

### The effect of PRL on macrophages

Macrophages are cells of the innate immune response located throughout the body. They detect, ingest, and degrade pathogens, cell debris, immune complexes, and apoptotic cells. They have a role in maintaining homeostasis, but at the same time, they may contribute to the pathogenesis of inflammatory and autoimmune diseases (102).

The expression of PRLR on the surface of macrophages indicates that PRL is involved in regulating the function of these cells (103). These findings are also supported by the observation that PRL can modulate the phagocytic and oxidative pathways in macrophages of healthy female rats (104). Ortega et al. have discovered that high concentrations of PRL could stimulate peritoneal macrophage phagocytosis and that PRL could modulate phagocytic processes exhibited by peritoneal macrophages, such as chemotaxis and microbicide capacity (105).

### The relationship between PRL, macrophages, and SLE

Studies in SLE patients and animal models have shown multiple alterations in the activation state and secretory function of circulating and tissue-infiltrating macrophages (106). Critically, this defect may explain the ineffective elimination of apoptotic cells by macrophages in SLE (107).

Macrophages activated in the presence of IFNγ and lipopolysaccharide are classified as proinflammatory M1 macrophages. In contrast, M2 macrophages, induced by IL-4 and IL-13, are anti-inflammatory (108). In SLE, the M1 to M2 ratio is altered. The inflammation observed during the development of lupus nephritis is characterized by an abnormal polarization of the M1/M2 commitment producing predominantly M1 macrophages. These macrophages infiltrate the kidneys and cause autoimmune damage (109). In a study comparing patients with active and inactive SLE, the upregulation of M1-associated genes was detected in active disease, while M2-associated genes were upregulated in clinically inactive patients (110) (**Figure 3**).

Svenson et al. have been the first to describe that macrophages exhibited reduced phagocytosis in approximately 50% of SLE patients. These defective cells also expressed low levels of CD44, reducing their adherence and affecting the clearance of apoptotic cells (111, 112). This inefficient clearance of apoptotic debris leads to the accumulation of autoantigens in GCs, causing the breakdown of B cell tolerance, the generation of plasma cells producing antinuclear antibodies, and the development of autoimmunity (113).

According to Majumder B’s findings, PRL might favor tumoricidal macrophages and drive Th-1 responses (114), inducing CD4 T cells to secrete cytokines, such as IFNγ, which regulates inflammatory immune responses and could contribute to SLE pathogenesis. Prolactin elicited transcriptional regulation related to IRF1 activation in monocytes, significantly increasing the transcription of many genes that are important in the SLE IFN signature, further supporting the role of PRL in SLE disease pathogenesis (115).

### The effect of PRL on NK cells

NK cells are CD3-negative cytotoxic lymphocytes that participate in the control of infections *via* their cytotoxicity and production of cytokines and chemokines. NK cells can be divided into two subsets. The more prevalent cells express CD56 at low levels and are positive for the Fcγ III immunoglobulin receptor, CD16+. These cells are the most cytotoxic. The smaller subset is CD56-positive but CD16-negative. These cells have been implicated in the abundant production of immunoregulatory cytokines (116). NK cells play a fundamental role in immune surveillance and elimination of damaged or autoreactive cells.

It is known that PRL specifically stimulates the development of CD34+ erythroid and myeloid progenitor cells (117). The PRLR signaling in CD34+ myeloid progenitors results in the activation of proinflammatory factors, such as IL-15, promoting the development of CD56+ NK cells (118). Interestingly, NK cells stimulated *via* the CD16 immunoglobulin receptors synthesize a peptide similar to PRL, suggesting that this hormone can activate NK cells in an autocrine manner (119). Hyperprolactinemia has been linked to NK cell activation and Th1-type cytokine induction in chronic inflammation (120). PRL synergizes with IL-15 to improve NK cell proliferation in a dose-dependent manner. Although PRL increases the cytotoxicity of IL-2- or IL-15-activated NK cells, PRL performs the function by upregulating perforin gene expression without influencing FasL in IL-2-stimulated NK cells (121).

### The relationship between PRL, NK cells, and SLE

In SLE patients, a reduction in the number and the cytotoxic function of peripheral blood NK cells has been demonstrated. This defect also resulted in reduced antibody-dependent cellular cytotoxicity (122). Morgane et al. have shown that the severity of the reduction in NK cell numbers correlated with disease activity in SLE. The remaining NK cells in SLE patients showed altered cytokine production and reduced degranulation in response to IL-15 and IL-18 stimulation. Furthermore, NK cells did not adequately regulate SLAMF1 and SLAMF7 expression in response to IL-2 and IL-12 in SLE. This affected NK cell-plasma cell interactions, leading to the accumulation of antibody-producing plasma cells, a characteristic feature of SLE (123).

On the other hand, the number of NK cells expressing PD1 and T-cell-induced negative regulator TIM-3 significantly increases in SLE. Furthermore, this increase correlates with worsened erythrocyte sedimentation rate and increased C-reactive protein and anti-dsDNA autoantibody levels. These changes also correlate with disease activity and severity (124).

Prolactin stimulates NK cell activation, which contributes to inflammation and the exacerbation of SLE. These findings support the fundamental role of PRL in the distorted development of NK cells and may be relevant to SLE development (**Figure 3**).

## Discussion

The pathogenesis of SLE is complex and multifactorial. The disease is often seen as the prototype of severe autoimmune inflammatory diseases characterized by the abnormal regulation of cellular immunity and the deposition of immune complexes. The clinical manifestations, symptoms, severity, and clinical responses show significant variability amongst SLE patients. Thus, no single mediator or pathway can adequately explain the complexities of its pathogenesis and clinical manifestations. However, the influence of PRL on the activity of multiple immune cell populations has been demonstrated, and a direct correlation is often seen between serum PRL levels and disease activity. These observations make further studies on the role of PRL in SLE and other autoimmune conditions imperative.

Although PRL is not a critical factor for the development of the immune response, it can affect the phenotype and function of various cells participating in both the innate and adaptive immune responses. Thus, PRL can be regarded as a multifunctional hormone. However, its role in immune regulation remains controversial. The variability of PRLR expression on the surface of macrophages, granulocytes, and T, B, and NK cells and the threshold PRL concentration necessary for optimal immune cell function are responsible for a complex picture that is difficult to interpret. This already complicated regulatory network is also affected by the microenvironment in which cells develop, the effect on cytokines and chemokines, and the production of PRL, or related proteins, by some inflammatory cells themselves. This results in a complicated system, where finding universal explanations for complex phenomena is very difficult. However, PRL undoubtedly has immunomodulatory effects. As discussed in this review, it is involved in the regulation of almost every aspect of the inflammatory/immune response, including proliferation and differentiation of immune cells, the expression of activation markers, the induction of T and B cell tolerance, the inhibition of apoptosis, antigen presentation, cytokine production, and the increased secretion of antibodies, particularly anti-DNA antibodies. Thus, the data presented in this review clearly show the relevance of PRL in autoimmunity, particularly due to its role as a modulator of both innate and adaptive immune responses (**Table 1**). However, understanding the full relevance of PRL in the pathogenesis of SLE will require a significant amount of future work.

**Table 1:** Effects of PRL on immune system cells in SLE.

Cells	Effects	Contribution to SLE pathogenesis	References
T cells	PRL promotes T cell activation and proliferation by increasing the expression of activation molecules, such as CD25, CD69, and CD154.	As an important step in SLE development, PRL can act between antigen presentation and costimulatory signals.	(23–25, 40)
	Reduces Treg cell suppressor activity, promoting IFNγ secretion.	This could indicate the connection between the PRL signaling pathway and the progression of SLE.	(45)
	Increase the number and activation of Tfh OX40+IL21+	It promotes the formation of GCs and interferes with the induction of tolerance.	(52)
	Alters the Th1/Th2/Th17 balance.	PRL may be involved in the Th1/Th17 axis, which is linked to SLE pathogenicity.	(59)
B cells	Affect B cell development and maturation, interfering with B cell tolerance induction.	Induces a lupus-like phenotype in non-susceptible mice and increases serum anti-DNA antibody titers.	(69–72)
	In a CD40-CD40L-dependent process, it inhibited BCR-mediated apoptosis of transient B cells.	Decreases the threshold for the activation of anergic B-cells and the B-cell tolerance induction.	(72)
	PRLR activation may affect clones of immature autoreactive B cells *via* STAT3 and regulation of apoptosis resistance-related genes.	Favors survival of autoreactive clones.	(73)
	Favor the pronounced secretion of anti-DNA IgG antibodies.	Contributes to the formation of immune complexes and tissue damage	(75)
Dendritic cells	Promote activation of pDCs	The result is an elevated expression of IFNα, leading to impaired immune tolerance.	(77)
	Enhance the production of cytokines (IL-12, TNF-α, and IL-1β) and promotes differentiation and activation of DCs	PRL could stimulate self-antigen presentation.	(79, 80)
Neutrophiles	Alters the phagocytosis and the intracellular killing of pathogens by neutrophils. Affect neutrophil migration.	Defective clearance of apoptotic cells, leading to the production of autoantibodies and inflammatory progression in SLE.	(100, 101)
Macrophages	Stimulate phagocytosis, chemotaxis, and microbicide capacity. Increase secretion of cytokines and chemokines and the release of ROS.	Increases inflammatory immune response.	(104, 105, 114)
NK cells	Induces proinflammatory cytokines, such as IL15, and promotes the development of CD56+ NK cells. PRL synergizes with IL-15 to improve NK cell proliferation and upregulate perforin gene expression.	It enhances an inflammatory response.	(118–121)

## Author contributions

ML-H, LC-S and PS-S designed the study and contributed to writing the manuscript. AC-R supervised the study. All authors were involved in drafting the article or revising it critically for important intellectual content, and all authors approved the final version to be published.

## Funding

This work was supported by CONACYT (grant number A1-S-9789), and Instituto Mexicano del Seguro Social (grant number R-2018-785-074).

## Conflict of interest

The authors declare that the research was conducted in the absence of any commercial or financial relationships that could be construed as a potential conflict of interest.

## Publisher’s note

All claims expressed in this article are solely those of the authors and do not necessarily represent those of their affiliated organizations, or those of the publisher, the editors and the reviewers. Any product that may be evaluated in this article, or claim that may be made by its manufacturer, is not guaranteed or endorsed by the publisher.
